# Association of *SLC19A1* Gene Polymorphisms and Its Regulatory miRNAs with Methotrexate Toxicity in Children with Acute Lymphoblastic Leukemia

**DOI:** 10.3390/cimb46100685

**Published:** 2024-10-16

**Authors:** Vasiliki Karpa, Kallirhoe Kalinderi, Eleni Gavriilaki, Vasiliki Antari, Emmanuil Hatzipantelis, Theodora Katopodi, Liana Fidani, Athanasios Tragiannidis

**Affiliations:** 1 Laboratory of Medical Biology-Genetics, School of Medicine, Aristotle University of Thessaloniki, 54124 Thessaloniki, Greece; vasilikikg@auth.gr (V.K.); kkalinde@auth.gr (K.K.); katopodi@auth.gr (T.K.); sfidani@auth.gr (L.F.); 2 2nd Propedeutic Department of Internal Medicine, Aristotle University of Thessaloniki, 54642 Thessaloniki, Greece; 3 Pediatric & Adolescent Hematology Oncology Unit, 2nd Pediatric Department Faculty of Health Sciences, Aristotle University of Thessaloniki, AHEPA Hospital, S. Kiriakidi 1, 54636 Thessaloniki, Greece; vasoantari@gmail.com (V.A.); hatzip@auth.gr (E.H.); atragian@auth.gr (A.T.)

**Keywords:** acute lymphoblastic leukemia, methotrexate, pharmacogenetics, miRNAs, oncology, toxicity, *SLC19A1*

## Abstract

Methotrexate (MTX) is an anti-folate chemotherapeutic agent that is considered to be a gold standard in Acute Lymphoblastic Leukemia (ALL) therapy. Nevertheless, toxicities induced mainly due to high doses of MTX are still a challenge for clinical practice. MTX pharmacogenetics implicate various genes as predictors of MTX toxicity, especially those that participate in MTX intake like *solute carrier family 19 member 1* (*SLC19A1*). The aim of the present study was to evaluate the association between *SLC19A1* polymorphisms and its regulatory miRNAs with MTX toxicity in children with ALL. A total of 86 children with ALL were included in this study and were all genotyped for rs2838958, rs1051266 and rs1131596 *SLC19A1* polymorphisms as well as the rs56292801 polymorphism of miR-5189. Patients were followed up (48, 72 and 96 h) after treatment with MTX in order to evaluate the presence of MTX-associated adverse events. Our results indicate that there is a statistically significant correlation between the rs1131596 *SLC19A1* polymorphism and the development of MTX-induced hepatotoxicity (*p* = 0.03), but there is no significant association between any of the studied polymorphisms and mucositis or other side effects, such as nausea, emesis, diarrhea, neutropenia, skin rash and infections. In addition, when genotype TT of rs1131596 and genotype AA of rs56292801 are both present in a patient then there is a higher risk of developing severe hepatotoxicity (*p* = 0.0104).

## 1. Introduction

Acute Lymphoblastic Leukemia (ALL) is the most common malignancy in children, characterized by an abnormal accretion of immature B or T lymphocytes in the bone marrow [1]. Over the last 20 years, mortality rates in children with ALL have declined rapidly due to the improvements in therapy and nowadays the Event-Free Survival and Overall Survival are above 90 and 85%, respectively [2]. One of the most important chemotherapeutic regimens that has long been used in the treatment of ALL is Methotrexate (MTX) [3]. MTX is an antagonist of folic acid that implicates a variety of transporters in its mechanism of action and can interfere in many biological processes inside the cell [4]. It is usually administrated intravenously in doses that can vary depending on the protocol followed [5,6].

Despite its therapeutic effectiveness, toxicities induced by MTX are a considerable issue that can affect the treatment’s outcome [7]. The most common adverse events caused by MTX are emesis, hepatotoxicity, neurotoxicity, mainly oral mucositis, myelosuppression and nephrotoxicity [8]. Serious side effects are documented in three-quarters of the patients that are following treatment, usually with a high dose of MTX (HD-MTX), resulting in death in 1–3% of them [5].

During the last few years, the association between MTX’s toxicity and genes has been extensively investigated by a large number of studies. Among the genes that are thoroughly researched are the ones that participate in MTX’s pathway inside the cell [2]. *solute carrier family 19 member 1 (SLC19A1)*, also known as *Reduced Folate Carrier 1 (RFC1)*, is a protein located in the membrane of the cells that plays an important role in the MTX intracellular transport pathway [9]. A number of different single-nucleotide polymorphisms (SNPs) in *SLC19A1* have been investigated so far for their association with MTX-induced toxicities, as well as MTX’s efficacy [3]. One of the most well-studied gene variants is rs1051266, previously referred to as rs61510559 [3,9]. Rs1051266 is found in the second exon of *SLC19A1* and specifically in position 80, where a Guanine can be changed to Adenine. As a result, the amino acid histidine is replaced by an arginine in the transmembrane protein translated by *SLC19A1*. This substitution as a result causes the alteration of the transporter structure and consequently a modification in its function too [10]. Correspondingly, the *SLC19A1* protein’s affinity with MTX changes and this results in a greater amount of the drug remaining in the central circulation system [11]. Rs1051266 has been associated with MTX’s treatment outcome, adverse events induced by MTX as well as MTX’s plasma levels [9,12]. The exact mechanism that clarifies the development of hepatic toxicity, although there is also a lower capability of MTX influx in liver cells, may implicate the involvement of other transporter proteins which are highly expressed in hepatic tissue [11,13]. Rs2838958 as well as rs1131596 is also amongst the important SNPs that are investigated for their possible relationship with side effects caused by MTX [3,14].

While a greater number of studies give emphasis to the investigation of genes that code for proteins implicated in MTX intake and metabolism, recent data underpin the important role of non-coding miRNAs that take part in the regulation of these genes. For example, miR-5189 seems to regulate *SLC19A1* and consequently MTX transport inside the cells [15]. To date, only one study related MTX levels in the plasma to *SLC19A1* polymorphism rs56292801 [2], while the number of researchers that are interested in the analysis of miRNAs and their roles in altering gene expression has increased rapidly.

Prediction of treatment-induced toxicities is a crucial point of interest in clinical practice, as it can help accomplish better therapy results and higher quality of life in patients [14]. This study aims to evaluate the association between *SLC19A1* polymorphisms and MTX toxicity in children with ALL. In addition, it is the first to investigate the correlation between the rs56292801 SNP in the miR-5189 gene together with rs1051266, rs2838958 as well as rs1131596 and MTX-induced adverse events in the same population.

## 2. Materials and Methods

### 2.1. Patients

In the present study, 86 children under the age of 16 years (50 male and 36 female) diagnosed with ALL were included in the study group. Patients were admitted and underwent treatment for ALL in the Hematology-Oncology Unit of the 2nd Pediatric Department Faculty of Health Sciences in AHEPA Hospital, Thessaloniki, Greece. Doses were administered according to the following protocols during the study period: ALL BFM 95 and BFM ALLIC 2009.

A written informed assent and/or consent form was signed by all patients aged >12 years, patient parents or guardians before the sample collection. This study was approved by the Ethics Committee of Aristotle University Medical School of Thessaloniki and was conducted conforming to the current version of the Helsinki Declaration.

### 2.2. Clinical Data and Toxicity Measurement

Patients underwent a short-term follow-up (48, 72 and 96 h after the administration of HD-MTX) in order to evaluate the presence of MTX-associated adverse events. The side effects of HD-MTX were classified into three categories: hepatotoxicity, mucositis and other side effects. Other side effects included nausea, emesis, diarrhea, neutropenia, skin rash and infections. Information on relapse or death was also collected.

The evaluation and grading of oral mucositis was performed according to the WHO scale, while the severity of drug-induced hepatotoxicity was assessed using the criteria shown in Table 1 [16]. For the statistical analysis, patients were further categorized into 2 groups: patients without or with mild hepatotoxicity (grades 0 or 1) and those with severe hepatotoxicity (grades 2, 3 or 4). Regarding age, patients were classified into 5 age groups: younger than 5 years, between 5 and 10 years old, 10 to 15 years old and older than 15 years.

### 2.3. Genotyping

Using phlebotomy, 5 mL of peripheral blood was collected into blood collection tubes with EDTA. Genomic DNA was extracted from peripheral blood white blood cells using the Canvax HigherPurity™ Blood DNA Extraction Kit. Genotyping of the SNPs was performed using Polymerase Chain Reaction (PCR) and the Restriction Fragment Length Polymorphism (RFLP) method.

PCR was performed for the amplification of rs2838958, rs1051266 and rs1131596 *SLC19A1* polymorphisms as well as the rs56292801 polymorphism of miR-5189 on the Eppendorf Mastercycler EP Gradient S PCR Thermal Cycler 5345. The cycling conditions used were as follows: initial denaturation at 95 °C for 5 min, 35 cycles of 30 s at 95 °C, 30 s at 65 °C decreasing to 0.4 °C in each cycle and 1 min at 72 °C. The final extension was set at 72 °C for 10 min and the final hold at 4 °C.

To determine the rs2838958 polymorphism, the following set of primers were used: Forward primer 5′-CAA CCT GGC CAT TCC CCT TC-3′ and Reverse primer 5′-GAG TGG TGG GCC TTA CAA CC-3′. For the amplification of the relative region encompassing the rs1051266 gene polymorphism, the set of primers used were as follows: Forward primer 5′-CCC TCC TTC CAG GCA CAG-3′ and Reverse primer 5′-CAG GAG GTA GGG GGT GAT GA-3′. For the genotyping of the rs1131596 gene polymorphism, the set of primers used were as follows: Forward primer 5′-CTC GTT TTG CGG GGT AGG GA-3′ and Reverse primer 5′-GCC ATG AAG CCG TAG AAG CAA-3′. Lastly, genotypes of the rs56292801 polymorphism of miR-5189 were defined using the following set of primers: 1.2 μL Forward primer 5′-TAG TTC CCA TCC TGG ACC CTG-3′, 1.4 μL and Reverse primer 5′-TTG TGA AAC TCG CTG TTC CC-3′. The length of each PCR product is as follows: 166 bp for rs2838958, 220 bp for rs1051266, 234 bp for rs1131596 and 247 bp for rs56292801 gene polymorphism.

PCR products were digested overnight using the Bsu36I, DraIII, HpyCH4III and EcoO109I Restriction Enzymes, respectively, for the above polymorphisms and the DNA fragments resulted from the digestion were separated by electrophoresis on a 4% agarose gel stained with ethidium bromide. Ultraviolet light was used for the visualization of DNA. The size of the DNA fragments after digestion and the genotype that corresponds to each fragment are shown in Table 2 for all the studied gene polymorphisms.

### 2.4. Statistical Analysis

The statistical analysis in this study was performed with the use of SPSS 29.0 statistical software. For continuous variables, a normality test was conducted using the Kolmogorov–Smirnoff test (N > 50). For dichotomous variables, which have only two categories, a chi-square test was performed in order to define the association between the two variables. Furthermore, Roc analysis was used in order to calculate at what age and above patients presented hepatotoxicity.

Principal component analysis was also applied in order to represent the correlation of all variables through the matrix table. Last but not least, a logistic regression was implemented in order to calculate the relationship between one of two categories of a dichotomous dependent variable and one or more independent variables that could be either continuous or categorical. In all cases, a *p* value equal to or less than 0.05 shows a statistically significant correlation between variables.

## 3. Results

### 3.1. Patient Demographics and Clinical Characteristics

Of the 86 patients recruited, 50 (58.1%) were male while 36 (41.9%) were female. The majority of the childhood patients (73 of 86) were diagnosed with B-cell ALL, while the rest of them were diagnosed with T-cell ALL. The age of the patients ranged between 4 months old and almost 17 years old. When it comes to the risk, 34 of the children were characterized as standard-risk, 37 as medium- and 15 as high-risk patients.

With regard to MTX toxicity, 59 (68.6%) of the 86 patients presented adverse events due to therapy with MTX, while 27 (31.4%) remained free of side effects. The frequencies of the specific adverse events induced by MTX are presented in Table 3.

### 3.2. Association between SLC19A1 Gene Polymorphisms Individually and MTX-Induced Adverse Events

In the present study, 86 patients were genotyped for rs2838958, rs1051266 and rs1131596 *SLC19A1* polymorphisms as well as the rs56292801 polymorphism of miR-5189. The genotype distribution for all the studied polymorphisms in patients that developed side effects is shown in Table 4. When mucositis, as well as other adverse events, was compared with any of the polymorphisms individually, no statistically significant correlation was found. When it comes to hepatotoxicity, logistic regression analysis demonstrated a significant association between the rs1131596 *SLC19A1* polymorphism and hepatotoxicity (*p* = 0.03). Specifically, patients with the CT genotype present a higher risk of developing severe hepatotoxicity.

### 3.3. Association between SLC19A1 Gene Polymorphisms in Combination and MTX-Induced Adverse Events

In order to analyze if there was an association between the presence of specific genotypes in each of the polymorphisms studied and MTX-induced adverse events, principal component analysis was conducted. The results indicate that when the TT genotype of rs1131596 and AA of rs56292801 are present simultaneously in a patient there is a statistically significant risk of developing severe hepatotoxicity (*p* = 0.0104) (Table 5). There is no significant difference between specific genotypes of all the four polymorphisms studied together and MTX-induced toxicities.

### 3.4. Association between Age of ALL Diagnosis and Hepatotoxicity

**Figure 1:**
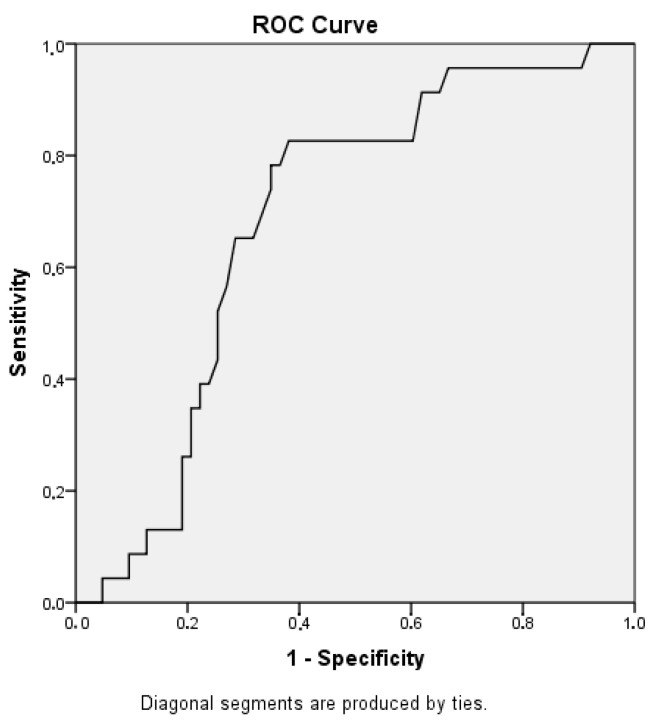
ROC curve: age of ALL onset versus hepatotoxicity development.

**Figure 2:**
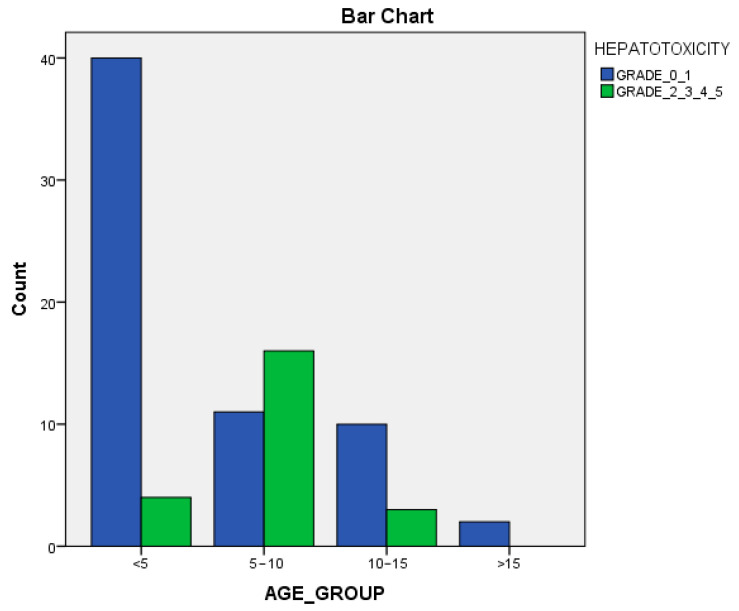
Bar chart: correlation between hepatotoxicity and age group.

ROC analysis was used in order to calculate at what age and above patients presented hepatotoxicity. The ROC curve that is presented in Figure 1 illustrates that the age of ALL onset is strongly associated with the development of hepatotoxicity (*p* = 0.009). To be more specific, childhood patients above 5 years and 1 month old seem to suffer from toxicity of the liver. Furthermore, chi-square tests showed that there is a statistically significant correlation between age group and hepatotoxicity, with the majority of the children that demonstrated hepatotoxicity belonging in the age group between 5 and 10 years old (Figure 2).

## 4. Discussion

One of the most commonly used chemotherapeutic regimens that is employed in the treatment of pediatric ALL is MTX [17]. Nevertheless, one of the major challenges of its use in ALL therapy is the development of MTX-related side effects including but not limited to liver toxicity, mucositis, neurotoxicity, myelosuppression and kidney injury [1,7,18]. The exact reasons why MTX causes toxicity are not clear yet [19]. During the last decades, many studies have investigated the association between gene polymorphisms and MTX-induced toxicity, supporting the view that the genetic profile of a patient can predict how they will respond to MTX treatment [2,20]. Furthermore, interactions between genes may have a key role in predicting treatment outcome, more than single genetic variants [20].

*SLC19A1*, or *RFC1*, is a transporter protein that is located in the membrane of mammalian cells, which is encoded by the homonymous gene [21]. It holds a significant role in importing folates inside cells, as well as chemotherapeutic drugs including MTX [21,22]. Polymorphisms of this gene seem to change this transporter’s structure and function, lowering its affinity with MTX and thus resulting in higher levels of MTX in the plasma [11]. For this reason, *SLC19A1* is among the genes that are well studied for their association with drug toxicity, but results are inconsistent [3]. Kotnik et al. in their study included 88 children and adolescents with ALL or non-Hodgkin lymphoma that were treated with HD-MTX. In their study, the rs2838958 *SLC19A1* polymorphism was found to be associated with mucositis that was developed after chemotherapy. Specifically, the TT genotype of rs2838958 was related to a higher risk of mucositis development than CC and CT genotypes [14]. Another study from Liu et al. concluded that the AA genotype of rs2838958 is linked with a less successful treatment outcome than AG and GG genotypes [23].

rs1051266 is one of the most investigated variants in *SLC19A1* [3,24]. Again, results remain controversial. Gregers et al. in their study concluded that patients that have the GG genotype of rs1051266 gene polymorphism are at higher risk of liver toxicity due to MTX, while bone marrow toxicity is more common in patients with the AA genotype [25]. In addition, a study by Kishi et al. showed that the A allele of rs1051266 is significantly related to gastrointestinal system impairment [26]. Esmaili et al. in their research concluded that *SLC19A1* rs1051266 GA genotype was related to a high risk of developing hepatic toxicity [24]. Salazar et al. claimed that the GG-genotype rs1051266 polymorphism is significantly correlated with the presence of hematopoietic toxicities [24,27]. On the other hand, studies from Chiusolo et al. and He et al. found no important associations between MTX toxicity and the rs1051266 gene polymorphism [28,29].

With regard to rs1131596, a few studies found no significant association between this variant and MTX side effects in ALL patients [23,30,31]. Specifically, Liu et al., in both studies conducted in 2016 and in 2017, respectively, found that there was no significant correlation between the rs1131596 gene variant and oral mucositis [23,30]. However, Grabar et al. showed that this polymorphism is related to MTX-induced adverse events in patients with Rheumatoid arthritis, suggesting that this variant could alter MTX toxicity and efficacy [32].

Until now, the main focus of researchers was genes that code for proteins. In the last few years, the interest has been directed to non-coding areas of the genome that can regulate the expression of genes, like miRNAs. These small-in-length single-stranded RNAs are implicated in the post-transcriptional regulation of genes, including those that participate in the transport of MTX [33]. In this way, miRNAs can regulate transporter protein function and, consequently, MTXs effectiveness and toxicity. To date, the number of the studied SNPs in miRNAs has increased rapidly, suggesting that they can be a promising new marker for the prediction of therapy-induced side effects [2].

One study from Iparraguirre et al. found that rs56292801 miR-5189 might affect *SLC19A1* transport gene regulation and could influence MTX levels in the blood, and by extension MTX toxicity in children with ALL [2]. To our knowledge, no study to date has examined *SLC19A1* polymorphisms in combination with its regulatory miRNAs for the development of MTX-induced side effects in childhood ALL.

In the present study, no significant association between rs2838958 and rs1051266 *SLC19A1* polymorphisms, as well as the rs56292801 polymorphism of miR-5189, with MTX-induced side effects (hepatotoxicity, mucositis and other adverse events) was found. When it comes to the rs1131596 *SLC19A1* polymorphism, logistic regression analysis demonstrated a significant association between the CT genotype and a higher risk of developing severe hepatotoxicity. When all four polymorphisms were studied in combination for the development of toxicities due to therapy, no significant difference between specific genotypes of all the polymorphisms studied together and MTX-induced toxicities was found. However, the results of the present study indicate that when the TT genotype of rs1131596 and AA of rs56292801 are present at the same time in a patient, there is a statistically significant risk of developing severe hepatotoxicity. In addition, according to the results of the present study, there is a statistically significant correlation between the age of ALL patients and liver toxicity, with the majority of the children that demonstrated hepatotoxicity belonging in the age group between 5 and 10 years old.

A limitation of this study was the relatively small number of patients recruited, so further research including a greater sample size is needed. However, all possible factors that could influence toxicity evaluation were taken into account. All of the patients that were enrolled came from the same geographic area, so they represent, in terms of origin, a genetically homogeneous population. In addition, all patients received medical care according to the same protocol, in one center. In this way, the process of clinical information collection from the patients was accomplished without important inconsistencies.

In conclusion, *SLC19A1* polymorphisms seem to be a promising predicting tool for MTX-induced toxicities, something that can improve patients’ quality of life via individualized therapy. In the last few years, the influence of miRNAs in treatment efficacy has gained researchers’ interest. A number of studies have revealed that polymorphisms in genes that code for miRNAs can alter the expression of genes involved in MTX metabolism and pathway inside the cells. In this way, miRNAs can affect therapy efficacy and as a consequence lead to better clinical outcomes. Nevertheless, additional research of MTX pharmacogenetics is needed as, to date, the results of a large number of studies remain inconsistent. Meta-analysis studies are necessary in order to provide biological explanations for the increased susceptibility of MTX-induced toxicity. Several genes are involved in the metabolism of MTX and in addition many miRNAs are implicated in the epigenetic regulation of *SLC19A1* genes, so not only single gene variants, but interactions between genes, could also be taken into account when it comes to drug-induced toxicity development. Physicians should be alerted and can minimize the risk of toxicity development by providing supporting care measures, especially in childhood patients, such as hydration, urine alkalization and other practices that can help in MTX’s elimination from the body [8,34]. The results of the present study could contribute to the understanding of the role that *SLC19A1* polymorphisms, as well as their regulating miRNAs, play in the development of toxicities due to MTX treatment. Additional studies are awaited to further elucidate the role of MTX pharmacogenetics in ALL therapy, aiming to facilitate the prediction of MTX-induced toxicities that can lead to therapy failures. 

## Figures and Tables

**Table 1:** Grading of hepatotoxicity severity.

Hepatotoxicity Grading	Definition
Grade 0	Without hepatotoxicity
Grade 1	SGOT or SGPT > 3ULN (often transient; many people adapt
Grade 2	SGOT or SGPT > 3ULN, TBL > 2ULN
Grade 3	Hospitalization
Grade 4	Liver failure
Grade 5	Death or liver transplantation

SGOT = Serum glutamic oxaloacetic transaminase, SGPT = Serum glutamate pyruvate transaminase, ULN= upper limit of normal, TBL = Total Bilirubin.

**Table 2:** Polymorphisms, size of the DNA products and DNA fragments after digestion in relation with the allele present.

Polymorphism	Gene Product Length	Alleles	Enzyme	Enzyme Digestion Products
rs2838958	166 bp	A	Bsu36I	166 bp
		G		133 + 33 bp
rs1051266	220 bp	A	DraIII	74 + 146 bp
		G		220 bp
rs1131596	234 bp	C	HpyCH4III	234 bp
		T		75 + 159 bp
rs56292801	247 bp	A	EcoO109I	68 + 113 + 66 bp
		G		68 + 15 + 98 + 66 bp

**Table 3:** Number of patients that developed adverse events after treatment with MTX.

Adverse Events	N = 86
Hepatotoxicity	32 (37%)
Mucositis	45 (52%)
Other (nausea, emesis, diarrhea, neutropenia, skin rash and infections)	32 (37%)

**Table 4:** Distribution of *SLC19A1* and miR-5189 polymorphisms in patients with MTX-induced side effects.

MTX Adverse Events	rs2838958			rs1051266			rs1131596			rs56292801		
	AA	AT	TT	GG	GA	AA	CC	CT	TT	AA	AG	GG
Hepatotoxicity	12 (14%)	13 (15%)	7 (8%)	16 (19%)	7 (8%)	9 (10%)	10 (12%)	7 (8%)	15 (17%)	4 (5%)	10 (12%)	18 (21%)
Mucositis	11 (13%)	22 (26%)	12 (14%)	21 (24%)	16 (19%)	8 (9%)	10 (12%)	21 (24%)	14 (16%)	5 (5%)	18 (21%)	22 (26%)
Other AEs	8 (9%)	10 (12%)	14 (16%)	13 (15%)	7 (8%)	2 (2%)	2 (2%)	9 (10%)	11 (13%)	3 (3%)	6 (7%)	13 (15%)

**Table 5:** Association between both rs1131596 and rs56292801 with the development of severe hepatotoxicity.

		rs56292801		
rs1131596		**AA**	AG	GG
	CC	*p* = 0.2627	*p* = 0.5221	*p* = 0.0703
	CT	*p* = 0.2627	*p* = 0.5221	*p* = 0.0703
	TT	*p* = **0.0104**	*p* = 0.3681	*p* = 0.7718

## Data Availability

Data are contained within the article.
